# Suppression of Tinnitus in a Patient with Unilateral Sudden Hearing Loss: A Case Report

**DOI:** 10.1155/2012/210707

**Published:** 2012-11-25

**Authors:** Alessandra Fioretti, Giorgia Peri, Alberto Eibenstein

**Affiliations:** ^1^Tinnitus Center, Department of Otorhinolaryngology, European Hospital, Via Portuense 700, 00149 Rome, Italy; ^2^Audin Clinic, Hearing Aid Center, Via Veneto 7, 00187 Rome, Italy; ^3^Department of Biotechnological and Applied Clinical Sciencies, University of L'Aquila, Via Vetoio, Coppito, 67100 L'Aquila, Italy

## Abstract

We describe a case of a 67-year-old woman with severe disabling right-sided tinnitus, mild hyperacusis, and headache. The tinnitus was associated with sudden right hearing loss and vertigo, which occurred about 18 months before. Magnetic resonance imaging (MRI) resulted in normal anatomical structures of the cochlea and of the cranial nerves showing a partial empty sella syndrome with suprasellar cistern hernia. Angio-MR revealed a bilateral contact between the anterior-inferior cerebellar artery (AICA) and the acoustic-facial nerve with a potential neurovascular conflict. Surgery was considered unnecessary after further evaluations. The right ear was successfully treated with a combination device (hearing aid plus sound generator). Shortly after a standard fitting procedure, the patient reported a reduction of tinnitus, hyperacusis, and headache which completely disappeared at the follow-up evaluation after 3, 6, and 12 months. This paper demonstrates that the combination device resulted in a complete tinnitus and hyperacusis suppression in a patient with unilateral sensorineural sudden hearing loss. Our paper further supports the restoration of peripheral sensory input for the treatment of tinnitus associated with hearing loss in selected patients.

## 1. Introduction

Sudden sensorineural hearing loss (SSHL) is defined as an acute deafness with abrupt onset, generally within 3 days of more than 30 dB hearing loss at three consecutive frequencies [1, 2]. The incidence of SSHL is 5–20 cases per 100.000 individuals in USA. There are many causes for sudden hearing loss which include infectious, circulatory, inner ear problems like Meniere's disease, and neoplastic, traumatic, metabolic, neurologic, immunologic, toxic, cochlear but in most patients the SSHL is idiopathic [3, 4]. The rate of spontaneous recovery exceeds the 2/3 of the cases. Due to the lack of a definite cause of SSHL, its treatment is still controversial and different protocols are suggested. The SSHL is often accompanied by tinnitus. Disorders of loudness tolerance like hyperacusis are often associated with tinnitus but the mechanisms are largely unknown. One model of tinnitus neurophysiology is based on the theory of a maladaptive attempts of cortical reorganization process due to peripheral deafferentation. In selected cases, the combination of open ear hearing aid and sound generator represents an efficient therapeutic tool for tinnitus, hyperacusis and hearing loss [5, 6]. 

## 2. Case Report

**Figure 1:**
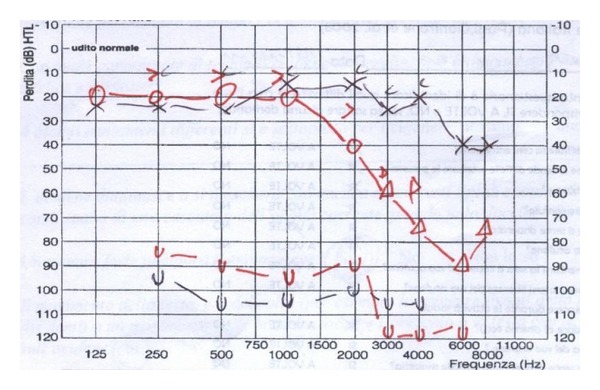
Pure tone audiometry testing revealed right-sided ski-slope hearing loss on high frequencies and mild hearing impairment on the high frequencies in the contralateral side. The presence of ULL of 85–90 dB in 3 frequencies revealed a mild right-sided hyperacusis.

**Figure 2:**
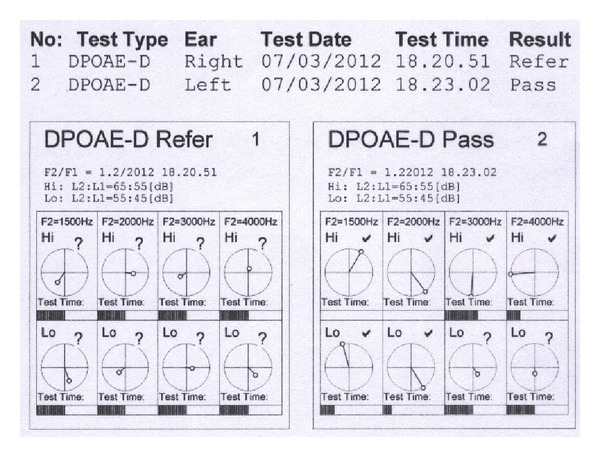
DPOAE were pass in the left ear and refer in the right ear.

**Figure 3:**
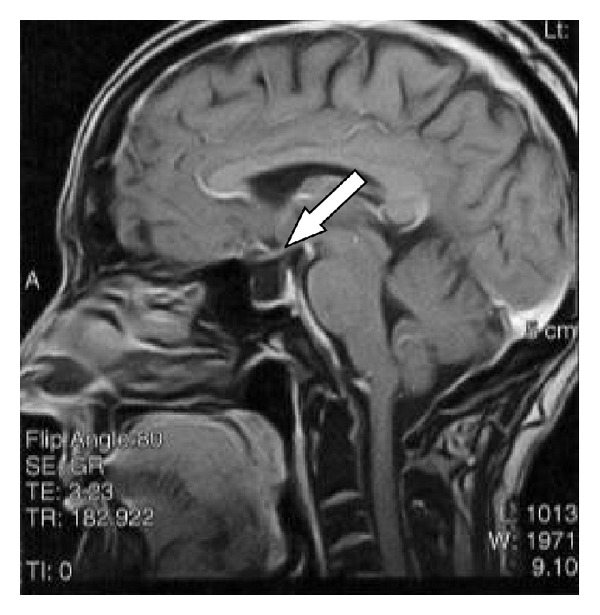
Partial empty sella syndrome with suprasellar cistern hernia on MRI.

A 67-year-old Caucasian woman came to our attention with complaints of severe disabling right-sided tinnitus, mild hyperacusis, and headache. Insomnia was also linked with the presence of tinnitus during the night. The tinnitus was associated with sudden right-sided hearing loss and vertigo, which occurred about 18 months before. Her hemogram, blood coagulation, blood sugar levels, lipid profile, and renal and liver functions were normal. Autoimmune markers and urine homocysteine tests were negative. In spite of different therapies (systemic steroids, antiviral, vasodilators) previously prescribed, there was no hearing improvement. Meanwhile the tinnitus worsened in the right ear (pitch: narrow band noise at 6 kHz). Tinnitus severity has been assessed with Tinnitus Handicap Inventory (THI) and visual analogue scales (VAS). Using a ten-point VAS, the tinnitus was rated by the patient according to loudness and annoyance. Insomnia and concentration problems due to tinnitus lead to severe disability and a poor quality of life (THI: 80, VAS: 9). Echo-Doppler of neck vessels was normal. Pure tone audiometry testing revealed right-sided ski-slope hearing loss on high frequencies and mild hearing impairment on the high frequencies in the contralateral ear (Figure 1). After the SSHL onset, the patient reported also headache accompanied by hyperacusis, described as an intolerance for ordinary environmental sounds. Hyperacusis was evaluated with the uncomfortable loudness level test (ULL) measuring the frequencies of 0.25 kHz, 0.5 kHz, 1 kHz, 2 kHz, 3 kHz, 4 kHz, and 6 kHz. Based on the presence of ULL of 85–90 dB in 3 frequencies, a mild right-sided hyperacusis was detected. DPOAE were present in the left ear and absent in the right ear (Figure 2). Magnetic resonance imaging (MRI) resulted in normal anatomical structures of the cochlea and the cranial nerves showing a partial empty sella syndrome with suprasellar cistern hernia in the absence of clinical signs and hormonal deficiency (Figure 3). Standard EEG was normal. During vestibular and neurological examination, spontaneous nystagmus and neurological deficits were not present. An SSHL when associated to vertigo must be considered as a potential sign for a widespread areas of infarction in the AICA territory [7, 8] and an angio-MR was performed. Angio-MR revealed a contact between the anterior-inferior cerebellar artery (AICA) and the acoustic-facial nerve with a potential neurovascular conflict. The neurosurgeon stated that surgery was unnecessary because he did not find a relationship with the tinnitus also taking in account that the contact was bilateral.

Even if the lipidic and glycemic blood profile of the patient resulted in normal values we suggested a low-carbohydrate diet, which improved her quality of life with the result of a reduction of headache and tinnitus. The patient reduced her weight loosing 12 Kg in two months without adverse effects. The insomnia was successfully improved with melatonin (3 mg/die).

**Figure 4:**
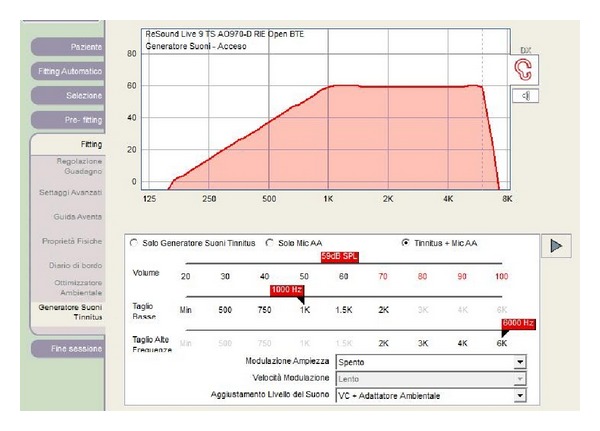
Fitting of sound generator in the combination device—the low cut filter was 1 kHz and the high cut filter was 6 kHz.

The right ear was treated with a combination device, which provides both hearing instrument features as well as a masking sound generator, used at least four hours per day. Initially it was activated, only the open fit hearing of the device and the process of monoaural fitting were effective with mild Noise Tracker, fixed directionality, and dual stabilizer DFS on. After 1 month the sound generator was activated and set to the mixing point so that the tinnitus and the sound generator stimulus blended together. The default noise setting for the generator was set to a broadband filtering setting with the flexibility of low and high cut controls to provide more individualized comfort. The low cut filter was 1 kHz and the high cut filter was 6 kHz, on the basis of audiological tests (Figure 4). The amplitude modulation was deactivated. Environmental steering was activated for the automatic volume control that adjusts the level of the white noise signal according to the listening environment. 

Shortly after a standard fitting procedure, the patient reported a reduction of her tinnitus, hyperacusis, and headache. The THI was significantly reduced at the follow-up evaluation after 6 months (THI: 20; VAS: 3) and 12 months (THI: 14; VAS: 2). After 6 months of sound therapy, the ULL test was normal and the hyperacusis disappeared as well as the discomfort related to the environmental noise. 

## 3. Conclusion

The decrease of afferent input caused by SSHL could induce tinnitus connected to central neural changes. Hyperacusis has received much less attention than tinnitus but it is a common and disabling symptom. This paper demonstrates that the combination device (hearing aid + sound generator) resulted in a complete tinnitus and hyperacusis suppression in a patient with unilateral SSHL. Our report further supports the restoration of peripheral sensory input for the treatment of tinnitus associated with hearing loss [9, 10] and the use of sound generator in the management of loudness perception disorders like hyperacusis. The effectiveness can be explained with the desensitization and the plastic reorganization of the central auditory nervous system due to this treatment, which has to be carefully assessed and individualized in selected patients. As evidenced by the THI scores and the ULL test significant improvement in tinnitus and hyperacusis perception is still present up to 12 months. In some patients, auditory disorders, including tinnitus, may be related to metabolic disorders, so we suggest a nutritional intervention program to control glucose and fat metabolism to support both pharmacological therapy and sound treatment.

## References

[1] Schreiber BE, Agrup C, Haskard DO, Luxon LM (2010). Sudden sensorineural hearing loss. *The Lancet*.

[2] Stachler RJ, Chandrasekhar SS, Archer SM (2012). Clinical practice guideline: sudden hearing loss. *Otolaryngology Head and Neck Surgery*.

[3] Chau JK, Cho JJW, Fritz DK (2012). Evidence-based practice: management of adult sensorineural hearing loss. *Otolaryngologic Clinics of North America*.

[4] Lin RJ, Krall R, Westerberg BD, Chadha NK, Chau JK (2012). Systematic review and meta-analysis of the risk factors for sudden sensorineural hearing loss in adults. *Laryngoscope*.

[5] Frachet B, Vormès E, Moyse D, Vasseur J (2004). Acoustic hearing aid with an integrated noise generator in hearing-impaired subjects with tinnitus. *Annales d’Oto-laryngologie et de Chirurgie Cervico Faciale*.

[6] del Bo L, Baracca G, Forti S, Moller AR (2011). Sound stimulation. *Textbook of Tinnitus*.

[7] Martines F, Dispenza F, Gagliardo C, Martines E, Bentivegna D (2011). Sudden sensorineural hearing loss as prodromal symptom of anterior inferior cerebellar artery infarction. *Journal for Oto-Rhino-Laryngology and Its Related Specialties*.

[8] Lee H (2012). Audiovestibular loss in anterior inferior cerebellar artery territory infarction: a window to early detection?. *Journal of the Neurological Sciences*.

[9] del Bo L, Ambrosetti U (2007). Hearing aids for the treatment of tinnitus. *Progress in Brain Research*.

[10] Moffat G, Adjout K, Gallego S, Thai-Van H, Collet L, Noreña AJ (2009). Effects of hearing aid fitting on the perceptual characteristics of tinnitus. *Hearing Research*.

